# Probabilistic modelling of material properties based on structural design and testing standards and its impact on the assessment of structural service life

**DOI:** 10.1038/s41598-026-42352-y

**Published:** 2026-03-19

**Authors:** Saeideh Faghfouri, Tânia Feiri, Marcus Ricker, Alfred Strauss

**Affiliations:** 1 Institute of Structural Engineering, BOKU University, Peter-Jordan-Straße 82, 1190 Vienna, Austria; 2 Chair of Structural Concrete, TU Dortmund University, August-Schmidt-Str. 8, 44227 Dortmund, Germany

**Keywords:** Sustainability, Structural concrete, Service life, Resilience, Conformity, Safety concepts, Engineering, Materials science

## Abstract

The reliability of engineering structures and infrastructure is a critical requirement to ensure a continuous functionality throughout their expected service life. The lifespan extension of concrete structures—especially those belonging to critical infrastructure—is vital to the sustainability and resilience of the whole built environment. This investigation explores the potential to extend the service lifetime of concrete structures by considering the role of modern design codes and conformity standards on concrete production in combination with reliability-based safety concepts applied to the resistance side. The study demonstrates how evaluation criteria derived from empirical concrete samples influence the service life of structures and, in consequence, the safety format established in international codes. The results suggest that by considering measures of concrete variability—as the coefficients of variation—and integrating them into a quality control system, hidden safety margins can be identified and, ultimately, activated to extend the service lifetime of structures. Therefore, this investigation contributes to the sustainable development of infrastructure ensuring that future demands on infrastructure can be met while maintaining high safety standards.

## Introduction

The calibration of partial safety factors is essential to ensure the safety of structures and technical systems. Partial safety factors for the resistance side account for uncertainties in material properties, load assumptions, and modelling approaches^1^. The partial safety factors of the semi-probabilistic safety concept are outlined in modern structural codes as in ÖNORM EN 1990:2023^2^ and depend on the quality of the base materials and their properties. Through the utilisation of partial safety factors, the prime requirement of structural engineering is to ensure a certain level of structural safety that is both technically justified and economically feasible. This structural safety level is typically measured by means of probability of failure ${P}_{f}$ and a corresponding reliability index $\beta$.

Over the last few years, numerous studies have been conducted in the field of structural reliability to adjust partial safety factors and activate hidden safety margins (or reserves). However, they mostly focus onextending the service lifetime of existing concrete structures. For example, the Joint Committee on Structural Safety (JCSS) has addressed this topic at its early stages^3^. A recent study^4^ discusses the reliability assessment of existing structures with emphasis on developments initiated by the JCSS or within the scope of the JCSS. For the assessment of concrete structures, the newly published *fib* Model Code 2020^1^ offers provisions for three key aspects: the residual capacity of existing structures without damage, the residual capacity of structures suffering deterioration, and the residual capacity of structures with noncompliant details^5^. By considering the experience gained in recent years in parallel with recent standardisation work in relation to EN Standards and the *fib* Model Code 2020, Orcesi et al. ^6^ offers the current state-of-the-art regarding partial safety factors for the assessment of existing structures. This investigation also addresses the differences between the utilisation of partial safety factors as provided in multiple national and international codes, adjusted partial factors derived for an individual case, and a reliability-based verification. In its turn, the *fib* Bulletin 86^7^ includes a set of probabilistic models for the most common basic variables influencing the resistance and load sides, discusses reliability-based methods, and stipulates target reliability indices $\beta$ to consider in load capacity assessments of existing highway bridges. Additionally, a novel methodology for quality-based service life assessment utilising reliability-based safety concepts was proposed in^8^. On the basis of this methodology, quality criteria can be associated with structural reliability by means of statistical models for specific parameters utilising a reliability index $\beta$. The extension of service lifetime of concrete structures based on semi-probabilistic considerations is offered in^9^. Increasing the service lifetime of civil structures in critical infrastructure while preserving the partial safety factor concept can significantly enhance their economic, ecological, and socio-ecological sustainability without compromising quality. This involves analysing the scatter values of material parameters—such as concrete compressive strength—by means of coefficients of variation or standard deviations as specified in design standards, quality control guidelines, and/ or sample surveys (e.g.,^2,10,11^).

The semi-probabilistic safety concepts of design standards (e.g., ÖNORM EN 1990:2013) and the probabilistic concepts of testing standards (e.g., ÖNORM B 4710-1:2018^12^) are based on distinct assumptions about the scatter of governing material parameters (e.g., concrete compressive strength). In principle, the scatter values provided in design standards can be significantly larger (i.e., more conservative) than the values established in testing standards. Under this assumption, a reduced scatter of such parameters enables the adjustment (i.e., reduction) of partial safety factors and, consequently, the identification of existing structural safety margins. A realistic verification of the actual performance of existing structures and the utilisation of safety margins can be assessed by means of reliability-based concepts and methods as the **F**irst or **S**econd **O**rder **R**eliability **M**ethod (FORM or SORM, respectively), among others  (e.g.,^4,13–20^).

This investigation sits in the context of a project conducted for an Austrian Infrastructure Operator aiming at utilising the potential of reliability-based approaches in combination with the positive influence of quality control provisions that are already in place to extend the service lifetime of their assets. A prime requirement of the project was to ensure the applicability of the proposed methodology. The starting point of this investigation focused on the design provisions on the resistance side. Two preliminary studies precede this manuscript:^21^ and^22^. By assuming that the (reference) partial safety factors established in ÖNORM EN 1990:2013 can be maintained, the goal of this investigation is to evaluate whether a semi-probabilistic partial safety concept can support the extension of the service lifetime of structures by means of a reduced scatter of material properties—in this case, concrete compressive strength. To this, the semi-probabilistic formulations offered in modern design standards^2^ are transformed into probabilistic formulations. Then, the most relevant basic variables involved on the resistance side—as it is the case of concrete compressive strength—are characterised including the quantification of the scatter underlying the semi-probabilistic method. Additionally, the criteria for limiting the scatter values of the basic variables—in this case by means of thresholds values defined for coefficients of variation (COV)—are assessed in accordance with quality control criteria established in ÖNORM B 4710-1:2018 and ÖNORM EN 1992-1-1:2021^23^. Then, the differences in the scatter of concrete compressive strength are analysed to evaluate their impact on the structural safety level by means of probability of failure ${P}_{f}$ and the corresponding reliability index $\beta$ and to determine whether an increase in the reliability index $\beta$ supports the extension of the service life of structures from 50 years (i.e., the default reference period established in design codes) to 150 years.

It should be noted that this study deliberately focuses on the initial reliability boundary conditions defined in current design and conformity frameworks. The reliability indices $\beta$ considered herein are, therefore, interpreted as code-based limit values at the beginning of the service life (time zero), representing the reliability level associated with design code calibration and the statistical assumptions embedded in conformity and quality control provisions. Consequently, time-dependent deterioration mechanisms (e.g., corrosion, carbonation, fatigue, or other degradation processes) are not explicitly modelled within the scope of the present contribution. Instead, the work investigates how the achievable reliability level under these initial conditions is influenced by the scatter in resistance properties (expressed through the coefficient of variation and supported by available production data). The extension of the framework towards time-variant reliability assessment and the explicit interpretation of $\beta$ for existing structures during operation ($\beta _{existing}$) will be addressed in subsequent research developments, particularly in the context of maintenance and preservation planning of civil structures.

This manuscript is organised as follows: Section “Overview on distinct quality assurance conformity standards” offers an overview on the distinct quality control in conformity standards for concrete production. Section “Methodological approach” details the methodology proposed for the analysis of measures of variability and section “Statistical analysis of concrete properties according to ÖNORM B 4710-1:2018” establishes the conformity criteria according to ÖNORM B 4710-1:2018. Section “Case study: statistical analysis of concrete properties from selected tunnel concretes” demonstrates how evaluation criteria derived from concrete samples can inform the adjustment of partial safety factors and, ultimately, support the extension of the service life of concrete structures. Section “Conclusions” lists the conclusions of this investigation and section “Limitations and recommendations” offers avenues for further investigations.

## Overview on distinct quality assurance conformity standards

In structural engineering, knowledge on concrete properties is critical to make inferences about the durability and the safety performance of civil structures (e.g.,^1,24–26^). Due to its heterogeneity, concrete properties, such as compressive strength, tensile strength, elastic modulus (or stiffness), permeability, among others, demonstrate variations in space and/ or in time (e.g.,^27–31^). According to^26,32^, variations in concrete compressive strength may originate from batch-to-batch variations and from within-batch variations (also known as within-test variations). The former results from changes to the ingredients or their proportions, water cementitious materials ratio (w/c), mixing, transporting, placing, sampling of the batch, consolidating, temperature, and curing. The later results from differences in the sampling of a batch sample, specimen preparation, curing, and testing procedures. To evaluate the extent of such variability and draw inferences about the quality of concrete, statistical methods or tools can be utilised already at the production stage (e.g.,^26,33^). In this context, modern structural codes, standards or best-practice guidelines include a wide range of conformity control provisions.

At the European level, the monitoring of concrete quality during production is regulated by the European Concrete Standard EN 206-1:2021^10^. Complementary national regulations may apply due to national climatic, material, political and, even, traditional differences^34^. In Austria, for example, ÖNORM EN 206-1:2021^35^ is connected to ÖNORM B 4710-1:2018^12^. In Germany, DIN EN 206-1:2021^36^ is linked to DIN 1045-2:2023^37^. Conversely, no significant differences exist in the regulatory framework of these two countries concerning the assessment of concrete variability during production. Such country-specific documents are in line with the base standard EN 206-1:2021 and establish two conformity control criteria for ready-mixed concrete depending on the production regime—i.e., initial production (IP) and continuous production (CP)—and sample size specifications (Table 1). First, the mean value of concrete compressive strength ${f}_{cm}$ must be estimated. Second, each individual concrete compressive value ${f}_{ci}$ must be determined through a compressive strength test (typically regulated in EN 12390-3:2019^38^)—regardless of whether the production sits in the IP or in the CP regime. Conformity is confirmed when both criteria are verified. Note that EN 12390-1:2021^39^ specifies the shapes (cube, cylinder and prism), dimensions and tolerances of cast concrete test samples. Yet, for each shape of a test specimen, the nominal size should be chosen to be, at least, three and a half times the maximum aggregate size (i.e., ${D}_{max}$ according to EN 206-1:2021). The minimum sampling frequency for conformity assessment varies according to the production regime (i.e., IP or CP), which in turn depends on the existing test results conducted during the assessment period. At the end of the IP phase, the standard deviation $\sigma$ of the population shall be estimated from, at least, 35 consecutive test results taken over more than three months. At the beginning of the CP period, this standard deviation $\sigma$ shall be utilised to assess the conformity for the first assessment period. At the end of the first assessment period and all subsequent periods, the empirical standard deviation ${s}_{n}$ shall be estimated by utilising the limits established in Table 2. If the empirical standard deviation ${s}_{n}$ does not change significantly, the current estimate of the standard deviation is utilised for the next assessment period. If the standard deviation has considerable changes, a new empirical standard deviation shall be determined from the last 35 consecutive test results and, then, shall be applied for the following assessment period.

**Table 1:** Conformity control criteria for concrete compressive strength according to^10,12,35–37^.

Production regime	Number of tests results ${n}$	Criterion 1:	Criterion 2:
		Mean value of concrete compressive strength ${f}_{cm}$ [N/$\hbox {mm}^{2}$]	Each individual result ${f}_{ci}$ [N/$\hbox {mm}^{2}$]
Initial production (IP) (number of test results ${n}$ < 35 )	3 $\le$ ${n}$ $\le$ 14	${f}_{cm}$ $\ge$ ${f}_{ck}$ + 4	${f}_{ci}$ $\ge$ ${f}_{ck}$ – 4
Continuous production (CP) (number of test results ${n}$ $\ge$ 35 )	${n}$ $\ge$ 15	${f}_{cm}$ $\ge$ ${f}_{ck}$ + 1.48 · $\sigma$	${f}_{ci}$ $\ge$ ${f}_{ck}$ – 4

$\sigma$: Standard deviation of a produced concrete sample

Note: The verification of conformity shall be based on test results obtained during the assessment period, which does not exceed the following two periods, depending on the testing frequency: For plants with a lower testing frequency (number of test results for concrete by properties less than 35 within three months), the assessment period shall include at least 15 results and a maximum of 35 consecutive results obtained over a maximum period of six months; For plants with a higher testing frequency (number of test results for concrete by properties at least 35 within three months), the assessment period shall include at least 15 results obtained over a maximum period of three months.

**Table 2:** Values to prove the empirical standard deviation ${s}_{n}$ according to^10,12,35–37^.

Number of test results ${n}$	Limits for ${s}_{n}$
15 to 19	0.63 · $\sigma$ $\le$ ${s}_{n}$ $\le$ 1.37 · $\sigma$
20 to 24	0.68 · $\sigma$ $\le$ ${s}_{n}$ $\le$ 1.31 · $\sigma$
25 to 29	0.72 · $\sigma$ $\le$ ${s}_{n}$ $\le$ 1.28 · $\sigma$
30 to 34	0.74 · $\sigma$ $\le$ ${s}_{n}$ $\le$ 1.26 · $\sigma$
35^a^	0.76 · $\sigma$ $\le$ ${s}_{n}$ $\le$ 1.24 · $\sigma$

^a^For more than 35 test results a condition based on the Chi-Squared distribution applies

In the U.S.A., the ACI CODE-318-25^40^—a structural code from the American Concrete Institute (ACI)—offers the basis of the building code requirements for the design and construction of concrete structures. According to this code, concrete mixture designs (i.e., including specifications for the expected performance for the mixture such as required strength, workability and durability, among others) must be submitted for approval. Typically, this submission is carried out by a project engineer, a materials and tests department of a government agency, or another designated authority. The goal is to demonstrate a very low risk of failing to meet the strength requirements before the concrete is utilised^33^. ACI PRC-214-11^32^ and ACI SPEC-301-20^41^—whose provisions are repeated in the Appendix of ASTM C94/C94M-23^42^—offer statistical-based methods to establish an average required strength ${f}^{\prime}_{cr}$ of a concrete mixture based on a specified nominal strength ${f}^{\prime}_{c}$ prescribed in design. To this, ACI SPEC-301-20 specifies two criteria: (i) every average of three consecutive tests equals or exceeds the specified strength ${f}^{\prime}_{c}$, and (ii) no strength test result falls below the specified strength ${f}^{\prime}_{c}$ by more than a specified value. By relying on these criteria, producers target an average required strength ${f}^{\prime}_{cr}$ greater than the specified strength ${f}^{\prime}_{c}$. This condition introduces a safety margin when proportioning and producing the concrete, which should follow the provisions of ACI PRC-211.1-22^43^. It should be highlighted that according to ASTM C31/C31M-23^44^ and ASTM C39/C39M-21^45^, the standard test for measuring the strength of concrete involves a compression test on cylinders cured for 28 days. Dimensions for the standard test cylinders are also defined. Yet, the primary requirement is that the diameter of the cylinder is, at least, three times the nominal maximum size of the coarse aggregate.

ACI PRC-214-11 requires the average required strength ${f}^{\prime}_{cr}$ to equal or exceed the specified strength in design ${f}^{\prime}_{c}$ by a multiple of the strength variation. This is selected to represent the percentage of tests allowed to be defective that finds a place according to specific criteria following the basic relationship:

1 $$\begin{aligned} {f}^{\prime}_{cr} = {f}^{\prime}_{c} + z \cdot s \end{aligned}$$

The term ${z}$ is the reliability factor which is selected to provide a sufficiently high probability that ${f}^{\prime}_{c}$ will be equaled or exceeded. In ACI PRC-214-11, a set of ${z}$ values are offered for various percentages of tests falling between the mean ± ${z}$ · ${s}$ (with ${s}$ being the empirical standard deviation of a concrete test series). In the U.S.A., the value ${z}$ equal to 2.33 is commonly utilised, which represents 1 $\%$ probability of falling below the specified strength ${f}^{\prime}_{c}$ .

For the strength results, ACI SPEC-301-20 offers two approaches: (i) computing a metric of dispersion from available historical strength test data or, (ii) in the absence of strength data, computing a conservative approximate metric of dispersion. For the metric of dispersion applied to available historical strength, the assessment can be made as follows^33^:

1. Submit a historical record of at least 30 consecutive tests on a mixture similar to that expected in the project in terms of materials, mixture proportions, quality control procedures, and climatic conditions. Here, the strength of the concrete mixture represented by the test records should be within 6.9 MPa (1000 psi) of the specified strength ${f}^{\prime}_{c}$ in design;
2. Submit the calculated empirical standard deviation for two jobs totalling 30 or more tests if a single past job with 30 tests cannot be found. In this case, the empirical standard deviations are calculated separately for each job and, then, statistically averaged. can only be utilised if the total number of tests from the two records is 30 or more.
3. Submit a record of 15 to 29 tests (from one job) if a similar mixture is available by calculating the standard deviation and applying the modification factor from Table 3. In this case, the test dataset should represent a single record of consecutive tests that span not less than 45 calendar days.

**Table 3:** Modification factors for the standard deviation to determine the required average strength ${f}^{\prime}_{cr}$ with sufficient historical data in accordance with the number of test results ${n}$^32^.

Number of test results ${n}$ ^a^	Modification factors for standard deviation
${n}$ $\le$ 15	Refer to Table 4
15	1.16
20	1.08
25	1.03
${n}$ $\ge$ 30	1.00

^a^Interpolate for intermediate number of tests

Then, the provisions of Table 4 apply. In the absence of sufficient strength test data (i.e., less than 15 test results), ACI SPEC-301-20 and ACI PRC-214-11 offer a more conservative approach, where the minimum ${f}^{\prime}_{cr}$ can be estimated by utilising the expressions in Table 4. For the control of concrete both in-situ and in laboratory, ACI PRC-214-11 offers ranges of reference values for the standard deviation and coefficients of variation. Reference values are available for batch-to-batch variations (Table 5) and for the within-batch variations.

**Table 4:** Minimum required average strength ${f}^{\prime}_{cr}$ with and without sufficient historical data^32^.

Specified concrete strength ${f}^{\prime}_{c}$ [MPa]	Minimum required compressive strength ${f}^{\prime}_{cr}$ [MPa]
*With sufficient historical data*	
${f}^{\prime}_{c}$ $\le$ 35	Maximum of:
	${f}^{\prime}_{cr}$ = ${f}^{\prime}_{c}$ + 1.34 · ${s}$
	${f}^{\prime}_{cr}$ = ${f}^{\prime}_{c}$ + 2.33 · ${s}$ – 3.5
${f}^{\prime}_{c}$ > 35	Maximum of:
	${f}^{\prime}_{cr}$ = ${f}^{\prime}_{c}$ + 1.34 · ${s}$
	${f}^{\prime}_{cr}$ = 0.90 · ${f}^{\prime}_{c}$ + 2.33 · ${s}$
*Without sufficient historical data*	
${f}^{\prime}_{c}$ < 21	${f}^{\prime}_{cr}$ = ${f}^{\prime}_{c}$ + 7.0
21 $\le$ ${f}^{\prime}_{c}$ $\le$ 35	${f}^{\prime}_{cr}$ = ${f}^{\prime}_{c}$ + 8.3
${f}^{\prime}_{c}$ > 35	${f}^{\prime}_{cr}$ = 1.10 · ${f}^{\prime}_{c}$ + 5.0

**Table 5:** Batch-to-batch variation: Standards of concrete control for ${f}^{\prime}_{c}$^32^.

${f}^{\prime}_{c}$ $\le$ 35: Standard deviation for different control standards [MPa					
Class of operation	Excellent	Very good	Good	Fair	Poor
Construction testing	< 2.8	2.8–3.4	3.4–4.1	4.1–4.8	> 4.8
Laboratory trial batches	< 1.4	1.4–1.7	1.7–2.1	2.1–2.4	> 2.4

${f}^{\prime}_{c}$ > 35: Coefficient of variation for different control standards (MPa)					
Class of operation	Excellent	Very good	Good	Fair	Poor
Construction testing	< 7.0	7.0–9.0	9.0–11.0	11.0–14.0	> 14.0
Laboratory trial batches	< 3.5	3.5–4.5	4.5–5.0	5.0–7.0	> 7.0

The standards from different regions worldwide, as those described in this Section, offer procedures and tolerances for testing the mechanical properties of concrete. This testing is essential to evaluate the variability of concrete and, ultimately, its quality and performance. Yet, there are variations in the specimen size, preparation, and curing requirements, as well as testing procedures and acceptance criteria among different standards^46^. Recently, Seyam & Nemes ^46^ stressed that it is essential to follow the appropriate standards and testing procedures to obtain accurate and representative test results given the differences in design format and, particularly, on the specifications for specimen preparation and curing. By following the relevant standards and procedures, it is possible to obtain consistent and reliable results that can be used to assess the quality and performance of concrete in distinct construction applications^46^.

## Methodological approach

### Thresholds for the coefficients of variation

As introduced in section “Introduction”, the concept of the probabilistic and semi-probabilistic partial safety format is described in European Standards as in ÖNORM EN 1990:2013^2^. Here, the design value ${X}_{d}$ of a material property (e.g., concrete compressive strength or yield strength) is determined from the characteristic value ${X}_{k}$ and the partial safety factor for the material property $\gamma _{m}$^2,11^:

2 $$\begin{aligned} {X}_{d} = \frac{{X}_{k} }{\gamma _{m}} \end{aligned}$$

ÖNORM EN 1990:2013 and ISO 2394:2015^47^ recommend to calibrate the design values ${R}_{d}$ and ${E}_{d}$ utilising the values of the variables ${R}$ (resistance) and ${E}$ (load effects) at the so-called design-point of the FORM method introduced in section “Introduction” (e.g.,^4,13–17,19,20^). The FORM method is an effective structural reliability approach that enables the computation of a failure probability ${P}_{f}$ of a structural component with respect to a limit state condition ${g}$(·):

3 $$\begin{aligned} {P}_{f} = {p}\,({g} \le 0) = \Phi (- \beta ) \end{aligned}$$

The failure probability ${P}_{f}$ corresponds to a reliability index $\beta$ through:

4 $$\begin{aligned} \beta \approx -\Phi ^{-1} ({P}_{f}) \end{aligned}$$

where $\Phi ^{-1}$ is the iintegral of the standardised Normal distribution. The $\beta$ value can be estimated for distinct reference periods by utilising the relationship between $\beta _{n}$ for a reference period of ${n}$ years and the reliability index $\beta _{1}$ for a reference period of 1 year^2^:

5 $$\begin{aligned} \Phi (\beta _{n}) = [ \Phi \, (\beta _{1})]^{n} \end{aligned}$$

While the reliability index $\beta$ is typically defined on the annual basis, i.e., $\beta _{1}$, a reference period of 50 years is normally considered in design. ÖNORM EN 1990:2013 offers target reliability indices $\beta$ according to target failure rates for the reference periods of 1 year and 50 years, and the consequence classes CC1 (low consequences), CC2 (medium consequences) and CC3 (high consequences). The methodology to determine the target $\beta$ value for a service life of 50, 100 and 150 years, which corresponds to a consequence class (CC), is comprehensively described, for example, in^8,9^. For a reference period of ${n}$ years and a specific consequence class, target $\beta$ values can be derived on the basis of Equation 5 (Table 6). It should be noted that $\beta _{1}$ refers to the required target reliability indices for the first year of the service life^9,22,48^.

**Table 6:** Target reliability indices $\beta$ for a structure depending on the consequence classes and the service lives (in years) alongside the thresholds for coefficients of variation (COV) under the assumption of a Normal distribution (N) and a Lognormal distribution (LN).

Class	Consequence	Service life (in years)	Target $\beta$ values	COV (N)	COV (LN)
			(Eq. 5)	(Eq. 11)	(Eq. 12)
CC2	$\hbox {Medium}$^a^	1	4.7	0.12	-
		100	4.8	0.12	0.19
		150	5.0	0.11	0.17
CC3	$\hbox {High}$^b^	1	5.2	0.11	-
		100	5.3	0.11	0.16
		150	5.4	0.10	0.15

^a^Medium consequence for loss of human life, economic, social or environmental consequences of failure consequences considerable (e.g., residential and office buildings)^2^.

^b^High consequence for loss of human, economic, social or environmental consequences of failure consequences very great (e.g., important bridges, public buildings)^2^.

Assumptions to apply Eqs. 11 and 12 : $\gamma _{m}$ = $\gamma _{C}$ = 1.5; $\alpha _{R}$ = 0.8; ${k}_{n}$ = 1.645

**Fig. 1:**
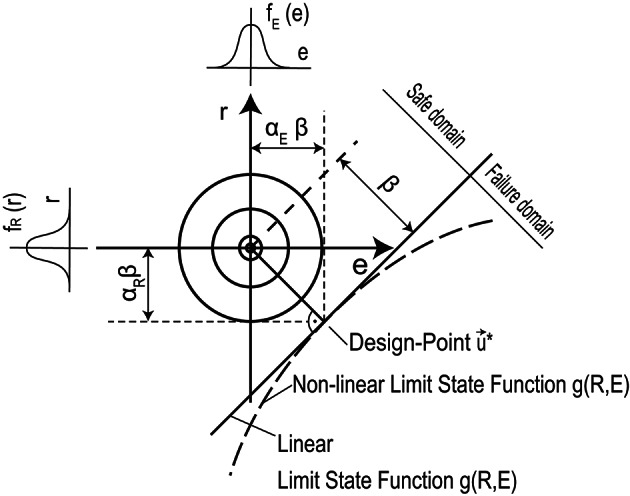
Illustration of the FORM method: Projection of the joint distribution density and the limit state function ${g(R,E)}$ = ${R}$ ${\displaystyle -\,}$ ${E}$ in the standard normal space (adapted from^49^).

The geometric definition of $\beta$ is symbolically illustrated in Fig. 1. To interpret Fig. 1, let’s assume that the limit state function ${g}$(·) is a function of the resistance ${R}$ and the load effect ${E}$ with ${g(R,E)}$ = ${R}$ – ${E}$. The cumulative distribution functions of the variables ${R}$ and ${E}$ are denoted by ${F}_{R}$(r) and ${F}_{E}$(**e**), respectively. For both random variables ${R}$ and ${E}$, the two distributions are characterised by the expected values $\mu _{R}$ and $\mu _{E}$ and the standard deviations $\sigma _{R}$ and $\sigma _{E}$. The coordinates of the FORM-based design-point, i.e. ${R}_{d}$ and ${E}_{d}$, are determined as:

6 $$\begin{aligned} {R}_{d}= & {F}_{R}^{-1} [ \Phi \, (\alpha _{R} \cdot \beta ) ] \end{aligned}$$

7 $$\begin{aligned} {E}_{d}= & {F}_{E}^{-1} [ \Phi \, (\alpha _{E} \cdot \beta ) ] \end{aligned}$$

with $\alpha _{R}$ and $\alpha _{E}$ being the sensitivity factors of the resistance ${R}$ and the load effect ${E}$, respectively^2^. If ${R}$ and ${E}$ are independent normal random variables, the design-point is defined as the point of the limit state surface closest to the average point in the space of normalized variables ${R}$/$\sigma _{R}$ and ${E}$/$\sigma _{E}$. By assuming that ${R}$ follows a Normal distribution, Eq. 6 can be rewritten as:

8 $$\begin{aligned} {R}_{d} = \mu _{R} - \alpha _{R} \cdot \beta \cdot \sigma \end{aligned}$$

According to^2^, $\alpha _{R}$ assumes the value of 0.8. With respect to this fixed sensitivity factor, it is possible to define analytical expressions for the partial safety factors $\gamma _{m}$ for a given target reliability level $\beta _{n}$ and a specific distribution function.

On the resistance side, the most relevant variable is material strength, which is generally described by a Normal or a Lognormal distribution (e.g.,^1,23,50^). Existing stochastic models for the description of concrete compressive strength utilise the assumptions of a Normal or a Lognormal distribution (e.g.,^14,51,52^). Yet, a Lognormal distribution is often superior for modelling material strength since it is bounded by zero, i.e., eliminating physically impossible negative strengths (e.g.,^53,54^). Typically, material strength is stochastically characterised by a long right tail, which the Lognormal distribution models more accurately than a Normal distribution.

The partial safety factor $\gamma _{m}$ can be established for a Normal distribution (N) (Eq. 9) and for a Lognormal distribution (LN) (Eq. 10).

9 $$\begin{aligned} \gamma _{m} \, \text {(N)}= & \frac{{X}_{k} }{{X}_{d}} = \frac{\mu _{X} \cdot \left[ 1 - {k}_{n} \cdot \text {COV}\,\text {(N)} \right] }{\mu _{X} \cdot \left[ 1 - \alpha _{R} \cdot \beta \cdot \text {COV}\,\text {(N)} \right] } \end{aligned}$$

10 $$\begin{aligned} \gamma _{m} \, \text {(LN)}= & \frac{{X}_{k} }{{X}_{d}} = \frac{\mu _{X} \cdot \text {exp} \left[ - {k}_{n} \cdot \text {COV}\,\text {(LN)} \right] }{\mu _{X} \cdot \text {exp} \left[ - \alpha _{R} \cdot \beta \cdot \text {COV}\,\text {(LN)} \right] } \end{aligned}$$

By utilising a reliability index $\beta$ for a corresponding consequence class and service life alongside a partial safety factor $\gamma _{m}$, the allowable scatter values for the concrete compressive strength are expressed as coefficients of variation (COV). These COV values can be estimated under the assumption that the strength values follow a Normally distribution (N) (Eq. 11) or a Lognormal distribution (LN) (Eq. 12):

11 $$\begin{aligned} \text {COV}\,\text {(N)}= & \frac{\gamma _{m} - 1}{\alpha _{R} \cdot \beta \cdot \gamma _{m} - {k}_{n} } \end{aligned}$$

12 $$\begin{aligned} \text {COV}\,\text {(LN)}= & \frac{\text {exp} (\gamma _{m})}{\alpha _{R} \cdot \beta - {k}_{n} } \end{aligned}$$

with the 5 $\%$ fractile being considered for the characteristic value of concrete compressive strength. By applying Eqs. 11 and 12, threshold values for the COV can be estimated for both distribution types and for two extended service lives: 100 years and 150 years (Table 6). It should be noted that according to^2^, ${k}_{n}$ is an unfavourable value for the characteristic value ${X}_{k}$ with unknown COV value. As it is later explained, by limiting the COV values of conformity test results—as it is proposed in Table 6—it ensures compliance with the target reliability indices $\beta$ for extended service lives (i.e., 100 years and 150 years).

### Statistical analysis of concrete compressive strength values for cubes and cylinders according to the design standard ÖNORM EN 1992-1-1:2021

As formulated in the design standard ÖNORM EN 1992-1-1:2021^23^—Sections 3.1.3 and 11.3.1—the distance between the 5 $\%$ fractile value ${f}_{ck}$ and the mean value ${f}_{cm}$ of concrete compressive strength is expressed through a constant value of 8 N/$\hbox {mm}^{2}$ resulting:

13 $$\begin{aligned} {f}_{cm} = {f}_{ck} + 8 \end{aligned}$$

As discussed in section “Overview on distinct quality assurance conformity standards”, EN 206-1:2021^10^ and ÖNORM B 4710-3:2023^55^ offer provisions to test strength in concrete samples. These standards indicate that cube samples should be stored under water. Since different storage conditions are considered in this investigation, the estimated strength for cube samples shall be multiplied by a factor of 0.92 for the classes up to C55/67 and by a factor of 0.95 for the classes higher than C60/75^12^. Thus, the 5 $\%$ fractile values of cube samples that are stored under environmental conditions (i.e., air) are determined as:

14 $$\begin{aligned} {f}_{ck,cube}^{*} = \frac{{f}_{ck,cube}}{0.92} \end{aligned}$$

The concrete compressive strength values obtained from the conformity tests on samples are affected by a factor ${k}_{ct}$ that accounts for high long-term loads and the duration of the load on the concrete compressive strength. According to^23^, the term ${k}_{ct}$ shall assume a value of 0.85 or 1.00 depending on the concrete class and on the duration of the load. For the sake of this investigation, ${k}_{ct}$ assumes a conservative value equal to 0.84. Under these assumptions, the 5 $\%$ fractile values for cube and cylinder samples were determined through Eqs. 15 and 16, respectively (Table 7).

15 $$\begin{aligned} {f}_{ck,cube}^{**}= & 0.84 \cdot {f}_{ck,cube}^{*}\,\,\,\,\, \text {with} \,\,\,\,\, {f}_{ck,cube}^{*} = \frac{{f}_{ck,cube}}{0.92} \end{aligned}$$

16 $$\begin{aligned} {f}_{ck}^{**}= & 0.84 \cdot {f}_{ck}^{*}\,\,\,\,\, \text {with} \,\,\,\,\, {f}_{ck}^{*} = \frac{{f}_{ck}}{0.92} \end{aligned}$$

**Table 7:** Concrete compressive strength values for cube and cylinder samples for different curing conditions—water curing, dry storage and in-situ strength—on the basis of ÖNORM EN 1992-1-1:2021^23^.

Classes	C20/25	C25/30	C30/37	C35/45	C40/50	C45/55	C50/60	C55/67	Conditions
*Cube samples*									
${f}_{ck,cube}$	25	30	37	45	50	55	60	67	water curing
${f}_{ck,cube}^{*}$	27.2	32.6	40.2	48.9	54.4	59.8	65.2	72.8	dry storage
$${f}_{ck,cube}^{**}$$	22.8	27.4	33.8	41.1	45.7	50.2	54.8	61.2	dry storage + in-situ strength
${f}_{cm,cube}$	33	38	45	53	58	63	68	75	water curing;
${f}_{cm,cube}^{*}$	35.2	40.6	48.2	56.9	62.4	67.8	73.2	80.8	dry storage
$${f}_{cm,cube}^{**}$$	30.8	35.4	41.8	49.1	53.7	58.3	62.8	69.2	dry storage + in-situ strength
*Cylinder samples*									
${f}_{ck}$	20	25	30	35	40	45	50	55	water curing
${f}_{ck}^{*}$	21.7	27.2	32.6	38.0	43.5	48.9	54.4	59.8	dry storage
$${f}_{ck}^{**}$$	18.3	22.8	27.4	32.0	36.5	41.1	45.7	50.2	dry storage + in-situ strength
${f}_{cm}$	28	33	38	43	48	53	58	63	water curing;
${f}_{cm}^{*}$	29.7	35.2	40.6	46.0	51.5	56.9	62.3	67.8	dry storage
$${f}_{cm}^{**}$$	26.3	30.8	35.4	40.0	44.5	49.1	53.7	58.2	dry storage + in-situ strength

**Table 8:** Statistical parameters of concrete compressive strength (cube and cylinder) according to ÖNORM EN 1992-1-1:2021^23^ for the concrete class C25/30 based on a Lognormal distribution for distinct curing conditions: water curing, dry storage and in-situ strength.

${q}$	0.95	1.00	1.05	1.10	1.15	1.20	1.25	1.30
Cube samples: Water curing $\rightarrow$ C25/30 with ${f}_{ck,cube}$ = 30 N/$\hbox {mm}^{2}$								
${q}$ · ${f}_{cm,cube}$	36.10	38.00	39.90	41.80	43.70	45.60	47.50	49.40
$\sigma$	3.90	5.20	6.60	8.00	9.40	10.90	12.50	14.00
COV	0.11	0.14	0.17	0.19	0.22	0.24	0.26	0.28
Cube samples: Dry curing $\rightarrow$ C25/30 with ${f}_{ck,cube}^{*}$ = 32.61 N/$\hbox {mm}^{2}$								
${q}$ · ${f}_{cm,cube}^{*}$	38.60	40.60	42.70	44.70	46.70	48.70	50.80	52.80
$\sigma$	3.90	5.30	6.80	8.00	9.80	11.20	12.70	14.50
COV	0.10	0.13	0.16	0.18	0.21	0.23	0.25	0.28
Cube samples: Dry curing + in-situ storage $$\rightarrow$$ C25/30 with $${f}_{ck,cube}^{**}$$ = 27.39 N/$$\hbox {mm}^{2}$$								
$${q}$$ · $${f}_{cm,cube}^{**}$$	33.60	35.40	37.20	38.90	40.70	42.50	44.20	46.00
$\sigma$	4.00	5.30	6.70	7.80	9.40	10.60	11.90	13.30
COV	0.12	0.15	0.18	0.20	0.23	0.25	0.27	0.29
Cylinder samples: Water curing $\rightarrow$ C25/30 with ${f}_{ck}$ = 25 N/$\hbox {mm}^{2}$								
${q}$ · ${f}_{cm}$	31.40	33.00	34.70	36.30	38.00	39.60	41.30	42.90
$\sigma$	4.10	5.30	6.60	7.80	9.10	10.40	11.80	13.20
COV	0.13	0.16	0.19	0.22	0.24	0.27	0.29	0.31
Cylinder samples: Dry curing $\rightarrow$ C25/30 with ${f}_{ck}^{*}$ = 27.2 N/$\hbox {mm}^{2}$								
${q}$ value · ${f}_{cm}^{*}$	33.40	35.20	37.00	38.70	40.50	42.20	44.00	45.8ß
$\sigma$	4.10	5.30	6.60	7.90	9.30	10.70	12.10	13.40
COV	0.12	0.15	0.18	0.20	0.23	0.25	0.28	0.30
Cylinder samples: Dry curing + in-situ storage $$\rightarrow$$ C25/30 with $${f}_{ck}^{**}$$ = 22.8 N/$$\hbox {mm}^{2}$$								
$${q}$$ · $${f}_{cm}^{**}$$	29.30	30.80	32.20	33.90	35.40	37.0	38.50	40.00
$\sigma$	4.30	5.40	6.50	7.70	9.00	10.20	11.50	12.80
COV	0.15	0.18	0.20	0.23	0.25	0.28	0.30	0.32

$\sigma$: Standard deviation; COV: Coefficient of variation

Note: The analysis was conducted by keeping the 5 $\%$ fractile value ${f}_{ck}$ constant while varying the mean compressive strength ${f}_{cm}$ by applying a factor from 0.95 to 1.30. For each assumed mean value, both standard deviation and COV were computed and adapted based on the Lognormal distribution using the fixed ${f}_{ck}$ as input. The iterative procedure is described in Appendix 1.

To evaluate the fluctuation of the mean values of the test samples compared to the mean values of the statistical population (i.e., expected value) defined in the design standard ÖNORM EN 1992-1-1:2021^23^, the quantiles ${q}$ of the mean values of compressive strengths where determined both for cubes and cylinders under the assumption of a Lognormal distribution.

The results listed in Table 8 refer to the 5 $$\%$$ fractile values of the concrete compressive strength for cube and cylinder samples for the class C25/30 stored according to distinct curing conditions. For water curing, the terms $${f}_{ck,cube}$$ and $${f}_{ck}$$ are used for cubes and for cylinders, respectively. For dry storage, the terms $${f}_{ck,cube}^{*}$$ and $${f}_{ck}^{*}$$ are used for for cubes and for cylinders, respectively. The effective concrete compressive strength is expressed through $${f}_{ck,cube}^{**}$$ and $${f}_{ck}^{**}$$, also for cubes and cylinders. These 5 $$\%$$ fractile values are utilised to evaluate the theoretical COV values for cubes and for cylinders.

**Fig. 2:**
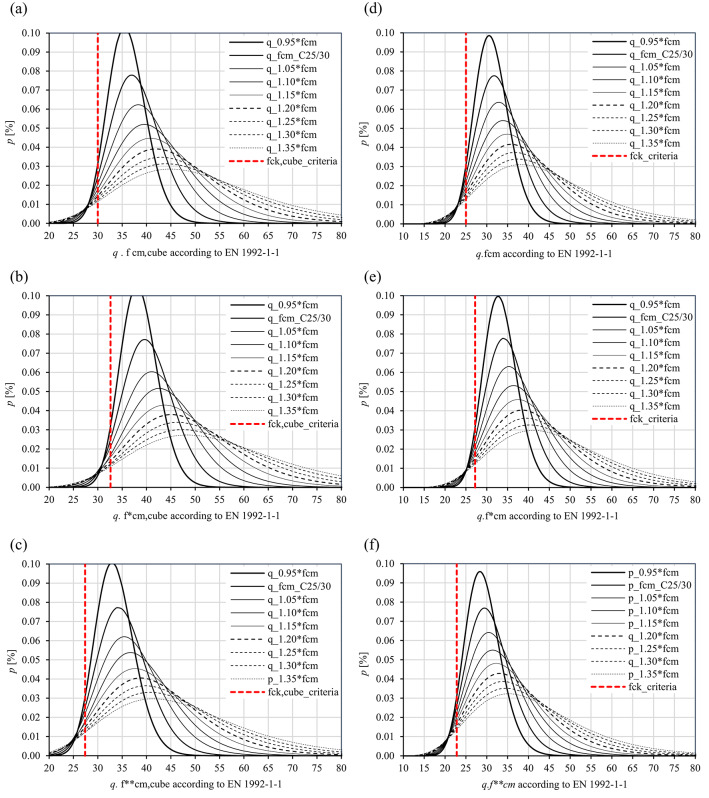
Probability density functions (PDF) of the Lognormal distribution for the concrete class C25/30 associated with 5 $$\%$$ fractile values (red lines) of concrete compressive strength according to ÖNORM EN 1992-1-1:2021^23^: (**a**) $${f}_{ck,cube}$$, (**b**) $${f}_{ck, cube}^{*}$$, (**c**) $${f}_{ck, cube}^{**}$$; (**d**) $${f}_{ck}$$, (**e**) $${f}_{ck}^{*}$$, (f) $${f}_{ck}^{**}$$.

The $${f}_{cm,cube}$$ and $${f}_{cm}$$ values sitting in the $${q}$$ range between 0.95 and 1.05 are considered plausible for correspondingly large samples; fluctuations beyond this range shall be separately assessed for each individual case. Complementary to the tabulated values, Fig. 2 demonstrates the Lognormal distribution functions of the lower fractile values $${f}_{ck,cube}$$, $${f}_{ck,cube}^{*}$$ and $${f}_{ck,cube}^{**}$$ for cubes, $${f}_{ck}$$, $${f}_{ck}^{*}$$ $${f}_{ck}^{**}$$ for cylinders alongside the $${q}$$-portion of the mean value of concrete compressive strength $${q} \cdot {f}_{cm,cube}$$ and $${q} \cdot {f}_{cm}$$. These parameters were utilised to determine the COV values alongside the design and the mean value of concrete compressive strength for a Lognormal distribution (Table 8).

As it is observed in Fig. 2 and from the values listed in Table 8, the COV values become larger with increasing ${q}$ values. It is relevant to underline that this variation simulates the effect of sample mean fluctuations in relation to the statistical population mean and it enables the evaluation of their influence on structural safety criteria. As it is demonstrated in Fig. 2, as the mean values increase, the distributions shift progressively to the right and, simultaneously, become broader. This behavior reflects the occurrence of overstrength in the material, leading to an increase in the overall variability. Increasing the mean strength values lead to a reduction in the probability of failure ${P}_{f}$, thereby, illustrating the beneficial effect of higher mean strength on structural reliability. In addition to capturing statistical variation, it can be argued that these distributions can be interpreted as representing the time-dependent development of concrete strength. Since concrete continues to gain strength over time due to ongoing hydration and environmental influences, an increase in the mean compressive strength can be associated with the time progression. The shift of the distributions toward higher strength values, therefore, reflects the natural gain of strength as it ages. This temporal evolution results in a corresponding decrease in the probability of failure ${P}_{f}$ neglecting the influence of deterioration processes. The fixed fractile value ${f}_{ck}$ acts as a reference threshold and the rightward shift of the distributions over time reflects an improvement in structural bearing capacity. Despite the associated increase in the COV value, the increasing mean strength results in a lower probability of failure ${P}_{f}$ and, therefore, enhanced structural reliability.

**Fig. 3:**
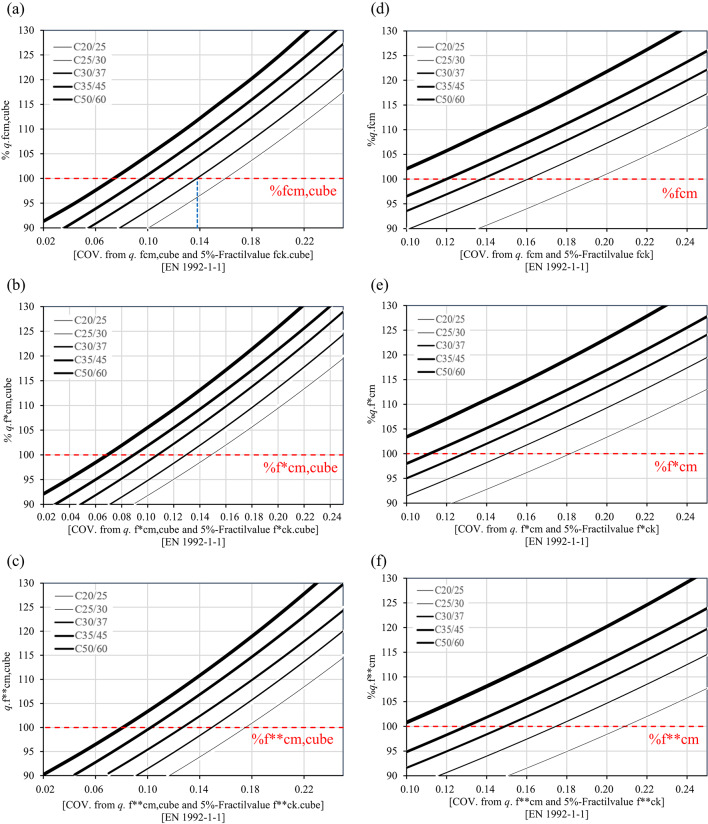
Variation of the mean value of concrete compressive strength (cubes) for the concrete classes C20/25, C25/30, C30/37, C35/45 and C50/60 according to ÖNORM EN 1992-1-1 ${vs.}$ COV values for distinct curing conditions: (**a**) and (**d**) water curing, (**b**) and (**e**) dry storage, and (**c**) and (f) dry storage with in-situ compressive strength.

Figures 3a–f offers a general overview of the trend followed by the COV values (horizontal axis) for the concrete classes C20/25, C25/30, C30/37, C35/45, and C50/60 both for cubes and cylinders. Figures 3a–c represent the data for cube compressive strength values, while Fig. 3d–f are based on cylinder compressive strength in accordance with ÖNORM EN 1990:2013^2^. On the vertical axis, the figures demonstrate that with an increasing value of ${q}$ (i.e., ${q}$ > 1.0), the COV values also increase since the lower 5 $\%$ fractile values ${f}_{ck}$ of the Lognormal distribution remain constant. For a ${q}$ value of 1.0, the COV values range between 0.07 and 0.17 for cubes and between 0.08 and 0.21 for cylinders. The results also indicate that only the class C20/25 fails to meet the criteria COV $\le$ 0.15 for a ${q}$ value of 1 for a service life of 150 years in the consequence class CC3 (Table 6). Therefore, considering a Lognormal distribution, the design standard ÖNORM EN 1992-1-1:2021 assumes COV values that support 150 years in CC3 for the concrete classes higher than C20/25. For all the concrete classes, the values suggest that extending the service lives to 100 and 150 years in CC2, as well as to 100 years in CC3, seems to be feasible.

It should be highlighted that this approach allows to evaluate how are the statistical parameters evolving when the mean strength increases independently of the fractile. The results indicate that the COV values increase as the mean strength also increases. Although a higher COV typically suggests large variability and, potentially, higher uncertainty, the mean values in these cases exceed the required design criteria including favorable overstrength. In this context, even with a higher COV value, the approach can still be considered reliable since the mean strength lies significantly above the characteristic limit defined according to ÖNORM EN 1990:2013. This suggests that a high COV value does not necessarily indicate reduced safety if the structural capacity, represented by the mean, remains above the threshold defined by the fractile.

Focusing on Fig. 3a for the concrete class C25/30, the vertical axis represents the mean compressive strength of concrete expressed as a percentage relative to the reference value, while the horizontal axis displays the corresponding COV values. The value 100 $\%$ on the vertical axis corresponds to the mean cube compressive strength ${f}_{cm,cube}$ defined in ÖNORM EN 1992-1-1:2021, as listed in Table 7. For this reference mean strength, the corresponding COV value on horizontal axis is 0.14, which aligns with Table 9 for a ${q}$ value of 1.0, where ${f}_{cm,cube}$ is equal to 38 MPa and the COV value is 0.14. From these figures it can be observed that the expected COV values for overstrength scenarios can be also retrieved, i.e., when the mean compressive strength exceeds the nominal value for five concrete strength classes ranging from C20/25 to C50/60.

Finally, it should stressed that overstrength (i.e., increased mean compressive strength) does not necessarily lead to higher reliability or longer service life. In practice, higher mean values are often accompanied by increased scatter (higher COV). Under conformity rules based on a lower-tail requirement (e.g., the 5 $\%$ fractile), this may even reduce the achievable reliability level. Therefore, this study does not interpret overstrength as inherently beneficial but rather analyses how an increase in mean strength may be associated with an increased COV while still satisfying the 5 $\%$ fractile criterion and maintaining the intended reliability level under the code-defined initial conditions. A systematic investigation of overstrength effects across different production regimes is beyond the scope of this paper and will be addressed in future work (see also^21^).

## Statistical analysis of concrete properties according to ÖNORM B 4710-1:2018

In this section, the thresholds for COV values for cube samples were defined according to ÖNORM B 4710-1:2018^12^ through a thorough statistical analysis. As listed in Table 1 (section “Overview on distinct quality assurance conformity standards”), two production regimes are established—initial production (IP) and continuous production (CP)—each with specific conformity control criteria (Table 9). Criterion 1 establishes a limit based on the required mean values of concrete compressive strength and Criterion 2 proposes a limit based on the required 5% fractile value for each individual result. In the following statistical analysis, Criterion 1 was analysed for both production regimes (i.e., IP and CP) with the goal to determine whether this criterion already governs the threshold for the COV values. In other words, the goal was to identify whether the COV values are lower than those presented in the design standard, as discussed in the previous section. Additionally, it was analysed whether the COV values—determined in accordance with the conformity criteria—fulfil the requirements to extend the service life of concrete structures to 100 and to 150 years.

**Table 9:** Criteria to define fractile and mean values of cube compressive strength according to ÖNORM B 4710-1^12^.

	Cond.	Initial Production (IP)	Continuous Production (CP)
Criteria 1: Mean values	1 2 3	$${f}_{cm,cube}$$ = $${f}_{ck,cube}$$ + 4 $${f}_{cm,cube}^{*}$$ = $${f}_{ck,cube}^{*}$$ + 4 $${f}_{cm,cube}^{**}$$ = $${f}_{ck,cube}^{**}$$ + 4	$${f}_{cm,cube}$$ = $${f}_{ck,cube}$$ + 1.48 · $$\sigma$$ $${f}_{cm,cube}^{*}$$ = $${f}_{ck,cube}^{*}$$ + 1.48 · $$\sigma$$ $${f}_{cm,cube}^{**}$$ = $${f}_{ck,cube}^{**}$$ + 1.48 · $$\sigma$$
Criteria 2: 5 $$\%$$ fractile values	1 2 3	$${f}_{ck,cube}$$ $${f}_{ck,cube}^{*}$$ = $${f}_{ck,cube}$$/0.92 $${f}_{ck,cube}^{**}$$ = $${f}_{ck,cube}^{*}$$ · 0.84	

1: water curing; 2: dry storage; 3: dry storage + in-situ strength

**Table 10:** Statistical parameters for the concrete compressive strength (cube) according to ÖNORM B 4710-1^12^.

Concrete	C20/25	C25/30	C30/37	C35/45	C40/50	C45/55	C50/60	C55/67
*Initial Production (IP)*								
${f}_{ck,cube}$	25	30	37	45	50	55	60	67
${f}_{ck,cube}^{*}$	27.2	32.6	40.2	48.9	54.4	59.8	65.2	72.8
$${f}_{ck,cube}^{**}$$	22.8	27.4	33.8	41.1	45.7	50.2	54.8	61.2
${f}_{cm,cube}$	29	34	41	49	54	59	64	71
${f}_{cm,cube}^{*}$	31.2	36.6	44.2	52.9	58.4	63.8	69.2	76.8
$${f}_{cm,cube}^{**}$$	26.8	31.4	37.8	45.1	49.7	54.2	58.8	65.2
*Continuous Production (CP)*								
${f}_{ck,cube}$	25	30	37	45	50	55	60	67
${f}_{ck,cube}^{*}$	27.2	32.6	40.2	48.9	54.4	59.8	65.2	72.8
$${f}_{ck,cube}^{**}$$	22.8	27.4	33.8	41.1	45.7	50.2	54.8	61.2
${f}_{cm,cube}$	29.4	34.4	41.4	49.4	54.4	59.4	64.4	71.4
${f}_{cm,cube}^{*}$	31.6	37.1	44.7	53.4	58.8	64.2	69.7	77.3
$${f}_{cm,cube}^{**}$$	27.3	31.8	38.2	45.5	50.1	54.7	59.2	65.6

$$^{*}$$ dry storage; $$^{**}$$ in-situ strength

**Table 11:** Statistical parameters for concrete compressive strength (cube) according to ÖNORM B 4710-1^12^ for the concrete class C25/30 based on a Lognormal distribution according to the concrete production regime and the conformity standard.

${q}$	0.95	1.00	1.05	1.10	1.15	1.20	1.25	1.30
*Initial Production (IP)*								
C25/30 with ${f}_{ck,cube}$ = 30 N/$\hbox {mm}^{2}$								
${q}$ value · ${f}_{cm,cube}$	32.30	34.00	35.70	37.40	39.10	40.80	42.50	44.20
$\sigma$	1.40	2.50	3.60	4.80	6.00	7.30	8.50	9.90
COV	0.04	0.07	0.10	0.13	0.15	0.18	0.20	0.22
C25/30 with ${f}_{ck, cube}^{*}$ = 32.61 N/$\hbox {mm}^{2}$								
${q} \cdot {f}_{cm,cube}^{*}$	34.80	36.60	38.40	40.30	42.10	44.00	45.80	47.60
$\sigma$	1.40	2.50	3.80	5.00	6.30	7.60	9.00	10.40
COV	0.04	0.07	0.10	0.12	0.15	0.17	0.20	0.22
C25/30 with $${f}_{ck,cube}^{**}$$ = 22.82 N/$$\hbox {mm}^{2}$$								
$${q} \cdot {f}_{cm,cube}^{**}$$	25.50	26.80	28.20	29.50	30.90	32.20	33.50	34.90
$\sigma$	1.70	2.60	3.50	4.40	5.40	6.40	7.40	8.50
COV	0.07	0.10	0.12	0.15	0.18	0.20	0.22	0.24
*Continuous Production (CP)*								
C25/30 with ${f}_{ck,cube}$ = 30 N/$\hbox {mm}^{2}$								
${q} \cdot {f}_{cm,cube}$	32.70	34.40	36.20	37.90	39.60	41.30	43.10	44.80
$\sigma$	1.70	2.80	4.00	5.20	6.40	7.70	9.00	10.30
COV	0.05	0.08	0.11	0.14	0.16	0.19	0.21	0.23
C25/30 with ${f}_{ck,cube}^{*}$ = 32.61 N/$\hbox {mm}^{2}$								
${q} \cdot {f}_{cm,cube}^{*}$	35.20	37.10	38.90	40.80	42.60	44.50	46.30	48.20
$\sigma$	1.40	2.60	3.90	5.10	6.30	7.70	9.10	10.50
COV	0.04	0.07	0.10	0.12	0.15	0.17	0.20	0.22
C25/30 with $${f}_{ck,cube}^{**}$$ = 27.39 N/$$\hbox {mm}^{2}$$								
$${q} \cdot {f}_{cm,cube}^{**}$$	30.20	31.80	33.40	35.00	36.60	38.20	39.80	41.40
$\sigma$	1.80	2.80	3.90	5.00	6.20	7.40	8.60	9.90
COV	0.06	0.09	0.12	0.14	0.17	0.19	0.22	0.24

$\sigma$: Standard deviation; COV: Coefficient of variation

$$^{*}$$ dry storage; $$^{**}$$ in-situ strength

Note: The analysis was conducted by keeping the 5 $\%$ fractile value ${f}_{ck}$ constant while varying the mean compressive strength ${f}_{cm}$ by applying a factor from 0.95 to 1.30.

For each assumed mean value, both standard deviation and COV were computed and adapted based on the Lognormal distribution using the fixed ${f}_{ck}$ as input. The iterative procedure is described in Appendix 1

**Fig. 4:**
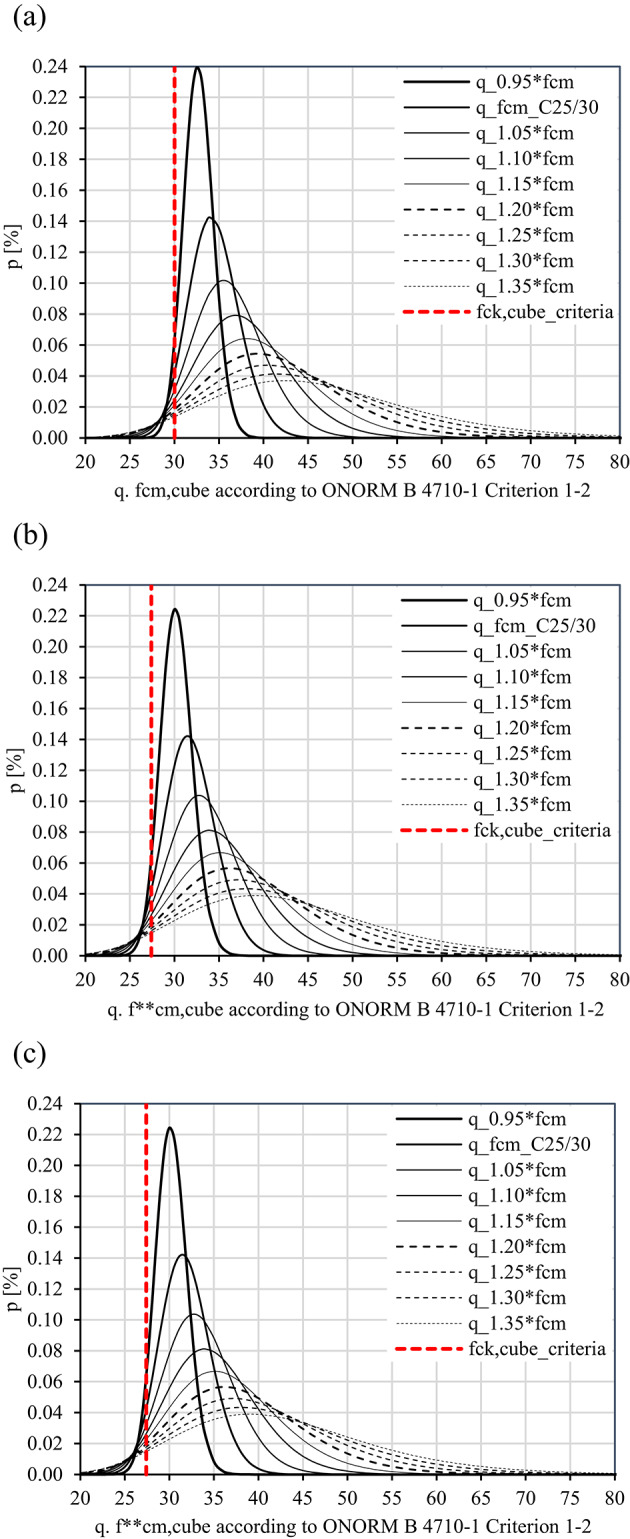
Probability density functions (PDF) of the Lognormal distribution for the concrete class C25/30 associated with 5 $\%$ fractile values (red lines) of concrete compressive strength according to the conformity standard ONORM B 4710-1^12^: (**a**) water curing (CP), (**b**) dry storage (CP), and (**c**) in-situ strength (CP).

Under the assumption of a Lognormal distribution, the 5 $\%$ fractile values of concrete compressive strength (red vertical line in Fig. 4) were calculated alongside the estimation of the mean values corresponding to these fractile values. The assessment was conducted for cube samples. In each assessment, eight concrete classes were considered for each specific production regime and the three different curing/ storage conditions, i.e., water curing, dry storage and in-situ strength (including dry storage) (Table 10).

The values in Table 11 list the statistical parameters determined for the concrete class C25/30 also for both production regimes under the assumption of a Lognormal distribution. Figure 4 displays the probability density functions for the CP regime. For the ${q}$ value of 1, the COV values range from 0.07 to 0.10. These values are significantly smaller than the counterpart COV values derived on the basis on the provisions of ÖNORM EN 1992-1-1:2021^23^ discussed in the previous section and the thresholds values established in Table 6.

**Fig. 5:**
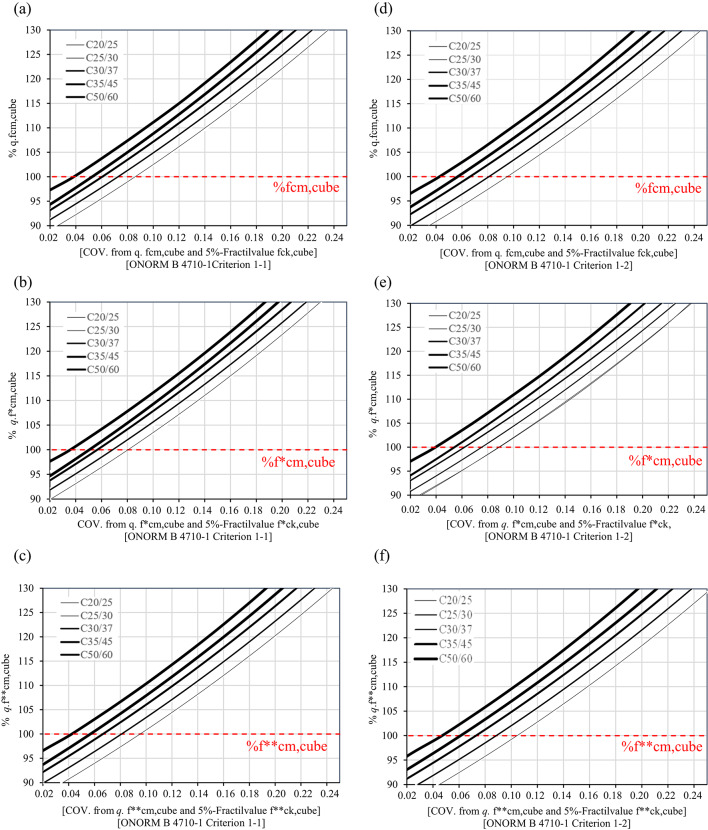
Variation of the mean value of concrete compressive strength (cubes) for the concrete classes C20/25, C25/30, C30/37, C35/45 and C50/60 according to the conformity standard ONORM B 4710-1^12^ ${vs.}$ COV values for distinct curing conditions: (**a**) water curing (IP), (**b**) dry storage (IP), (**c**) in-situ strength (IP), (**d**) water curing (CP), (**e**) dry storage (CP), and (**f**) dry storage with in-situ strength (CP).

Figure 5 demonstrates the trend of the COV values for five concrete classes in accordance with the two production regimes mentioned above. To interpret Fig. 5, a similar approach to that described for Fig. 3 can be utilised. Figures 5a–c refer to Criterion 1-1 established in ÖNORM B 4710-1:2018. Figures 5d–f correspond to Criterion 1–2 of the same standard. The values on the horizontal axis of Fig. 5 increase for the concrete compressive strengths for ${q}$ > 1 (vertical axis). Overall, the COV values range from 0.05 to 0.10 and are significantly smaller than the counterpart values derived according to ÖNORM EN 1992-1-1:2021. The diagrams also indicate that for all the concrete classes and for a ${q}$ value of 1, the COV values fulfil the criterion COV $\le$ 0.15 established for a service life of 150 years. These results suggest that the COV values allow for an increased service life to 150 years while maintaining the semi-probabilistic safety concept and the partial safety factors for the examined concrete classes.

## Case study: Statistical analysis of concrete properties from selected tunnel concretes

### General considerations

In this section, the above-described methodology was applied to a tunnel system of an Austrian Infrastructure Operator with the aim to estimate the reliability index $\beta$, as a measure of structural safety. The goal of this analysis is to demonstrate that the COV values of the concrete compressive strength measured on cube samples are lower than the threshold values established for the COV for a service life of 150 years and a consequence class CC3 established in Table 6.

### Dataset of concrete compressive strength values

### Analysis

**Fig. 6:**
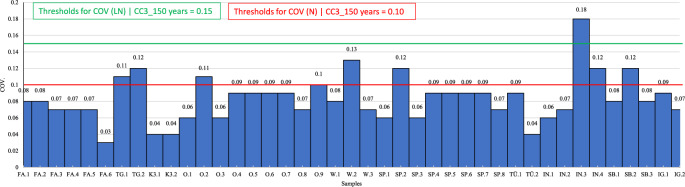
Variation of the COV values for all tunnel concrete samples.

For this analysis, multiple concrete samples with distinct concrete classes were analysed. The testing period of each sample is described alongside the concrete class. The variation of the COV values for all tunnel concrete samples are illustrated in Fig. 6. An extract of the samples (FA1 to FA6) is statistically characterised in Table 12. For example, the sample FA.1 corresponds to concrete class C25/30 and it was tested at 56 days or after 56 days. The stochastic behaviour of the cube samples produced at the tunnel was analysed through the mean values and the standard deviations enabling the estimation of the COV values of the samples under the assumption of a Lognormal distribution.

**Table 12:** Statistical parameters of the test samples produced at a specific tunnel location.

Samples	FA.1	FA.2	FA.3	FA.4	FA.5	FA.6
Concrete class	C25/30(56)	C25/30(56)	C25/30(56)	C25/30(56)	C25/30(56)	C25/30(56)
$\mu _{x}$	44.9	44.8	43.1	48.1	44.3	46.7
COV	0.08	0.08	0.07	0.07	0.07	0.03
${f}_{ck,cube,m}$	36.7	36.6	35.7	39.6	36.9	41
${f}_{ck,min}$	39.0	39.7	38.4	41.5	40.3	45.4
${f}_{min}$	38.4	39.1	37.7	41.1	39.8	45.3
No. samples	255	122	66	136	72	7

**Fig. 7:**
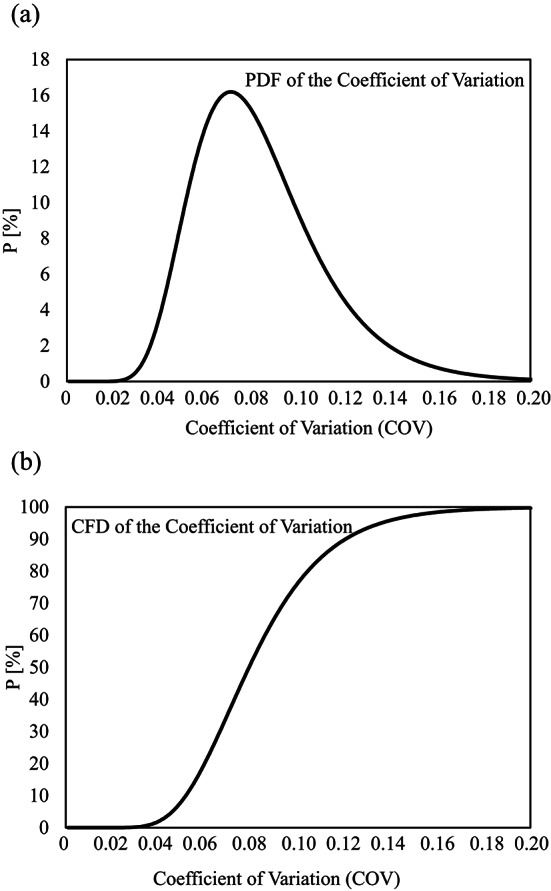
Coefficient of variation (COV) values for all the tunnel concrete samples assuming a Lognormal distribution: (**a**) Probability Density Function (PDF) and (**b**) Cumulative Function Distribution (CFD).

**Fig. 8:**
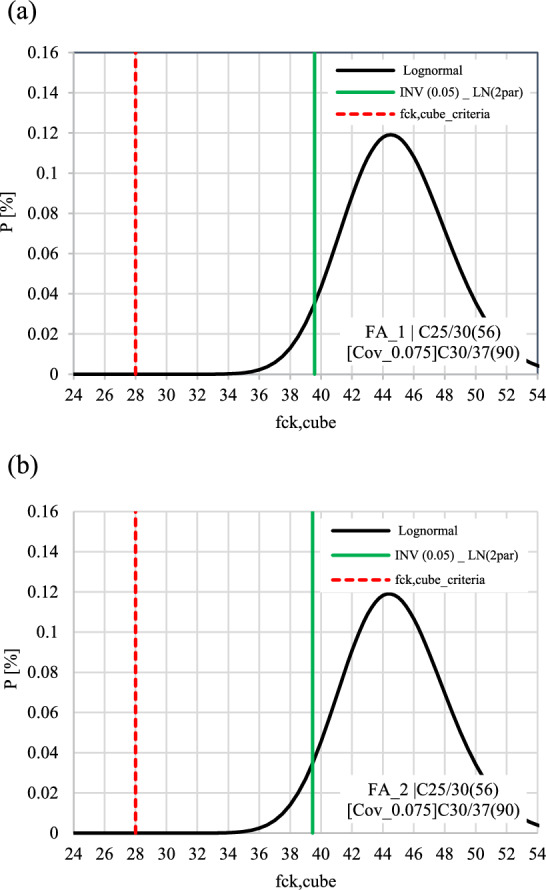
Probability density function (PDF) of the Lognormal distribution and corresponding ${f}_{ck}$ of the target concrete class for (**a**) Sample 1 and (**b**) Sample 2.

In Fig. 7, the probability density function (PDF) and the cumulative distribution function (CFD) were derived under the assumption of a Lognormal distribution. The variation of the COV values is described as follows: 70 $\%$ are below a COV of 0.10, 90 $\%$ are below a COV of 0.12, 95 $\%$ of the samples have a COV value below 0.15 and 99 $\%$ of the samples have a COV value below 0.17 (Table 6). This distribution is influenced by a COV of 0.18 for the inner shell concrete (Fig. 6). This suggests that the required thresholds values for the COV for the consequence class CC3 are attained and, thus, an increased service life to 150 years is feasible while maintaining the semi probabilistic safety concept and the partial safety factors of the design standards.

 The subsequent step aimed to assessed to which extent the calculated 5 $\%$ fractile values of the concrete compressive strength (measured on cube samples) exceed the 5 $\%$ fractile values specified in the design of the specific concrete classes. To this, the 5 $\%$ fractile values of the concrete compressive strength of samples extracted in various locations (as outlined in the design standard) were estimated according to Eq. 17 as a criterion for the compressive strength of the concrete samples (indicated by the red dashed line in Fig. 8). The critical values were determined based on ÖNORM B 4710-1:2018^12^ as:

17 $$\begin{aligned} {f}_{ck,cube,m}\,= & \, ({f}_{cm} - 1.48 \cdot \sigma ) \cdot 0.92 \end{aligned}$$

18 $$\begin{aligned} {f}_{ck,cube,min}\,= & \, ({f}_{cm} + 4) \cdot 0.92 \end{aligned}$$

where ${f}_{cm}$ is the mean value of the concrete compressive strength (cube samples) and ${f}_{ck,cube,min}$ is the minimum value of compressive strength (cube samples) at each location.

The 5 $\%$ fractile values of the actual cube compressive strength samples were estimated by assuming a Lognormal and utilising the software FReET^56^. An example of these calculations if offered in Table 12 and illustrated in Fig. 8 focusing on a specific tunnel location. The results suggest that the 5 $\%$ fractile values of the actual concrete compressive strength (green vertical line in Fig. 8) are significantly higher than the 5% fractile values of the concrete classes specified in the design procedure (red vertical line in Fig. 8).

## Conclusions

This manuscript investigates different safety formats at the design and conformity levels based on the statistical characterisation of concrete compressive strength. The detailed interactions between reliability, material statistical variability, and safety formats were investigated leading to the following conclusions:

- The influence of the COV on the reliability level and the corresponding service life estimates is explicitly reported in Table 6. The selected reference values (COV equal to 0.10 and to 0.15) are consistent with the probabilistic modelling framework underlying EN 1990 and should, therefore, be understood as normative, practically motivated by the reference levels rather than adjustable thresholds. The results demonstrate consistent trends across the investigated range of concrete classes, supporting the robustness of the derived conclusions.
- The results suggest that the two distributions assumed for the characterisation of concrete compressive strength—Normal distribution and Lognormal distribution—enable the extension of service lifetime of a structure (consequence class CC3) from 50 years to 150 years.
- Assuming a Lognormal distribution for concrete compressive strength enables a better performance than under the assumption of a Normal distribution. To guarantee the extension of service lifetime of a structure (consequence class CC3) to 150 years, a threshold value of 0,.10 is needed for the coefficient of variation under the assumption of a Normal distribution, while the threshold increases to 0.15 under the assumption of a Lognormal distribution.
- The analysis of the provisions in ÖNORM EN 1992-1-1:2021 suggest that only concrete class C20/25 fails to meet the limiting criteria established for these threshold values, in which a COV smaller than 0.15 does not guarantee a service life of 150 years also under the assumption of a Lognormal distribution.
- On the basis of a Lognormal distribution, the results indicate that the defined lower fractile values were not exceeded by more than 5 $\%$ and that they complied with the mean value. For the investigated concrete classes, the analysis revealed that the conformity test standard ÖNORM B 4710-1:2018—through its verification criteria for both initial and continuous production regimes—indirectly limits the COV to values lower than 0.10. This limitation enables an increase of the service life to 150 years while upholding the semi-probabilistic safety concept and the partial safety factors.
- The results also suggest that the actual limits for the COV values are slightly higher due to the increased mean value, constant fractile value and the use of a Lognormal distribution.
- Regarding the case study addressing the concrete compressive strength of cube samples taken from tunnel construction sites in accordance with the standard specifications, the results indicate that a Lognormal distribution offers a realistic representation of lower limit values. The application of the limit criterion below 0.15 discussed in this investigation seems feasible. The criterion meets the threshold value required for a consequence class CC3 and, thus, enables an extended service life of 150 years while maintaining the semi-probabilistic safety concept and the partial safety factors outlined in the design standards.

## Limitations and recommendations

From this investigation, a set of avenues was identified for future investigations:

- On the resistance side, the presented methodology can be extended and applied to a wider scope of concrete samples with the goal to assess whether smaller thresholds can be established for the COV values. To this, distinct concrete types can be investigated addressing distinct aggregates and mixtures from different geographic areas.
- Additional distribution types can be included in the methodology for the characterisation of concrete compressive strength. For example, the JCSS Probabilistic Model Code^50^ assumes that concrete compressive strength is characterised through a Log-student-${t}$ distribution. This distribution is commonly utilised to estimate the population parameters of small sample sizes. Since the Student-${t}$ distribution has a greater chance for extreme values than Normal distributions, they typically have heavier (or fatter) tails, which can be relevant for reliability-based studies.
- Previous investigations have demonstrated that Bayesian statistics are widely applied to update the distributional characteristics of material or mechanical properties of new or existing components (e.g.,^53,54,57–59^). When new or additional experimental data becomes available, it can be utilised to update prior knowledge on concrete compressive strength into a posterior belief through the Bayes’ Theorem. Consequently, these updated distributions can be taken into account when performing structural analysis, especially in case of structural reliability calculations. A methodology to update prior distributions of material properties is offered in^53^.
- The definition and consideration of more stringent quality control assessment beyond those offered in modern standards, i.e., tailor-made conformity criteria may be considered alongside their influence on the remaining service lifetime of structures as recommended in^60^. Principles for the control of quality and of acceptability of concrete are recommended in^61^.
- The extent to which the findings are transferable to other regulatory frameworks, namely regarding the international applicability of the proposed methodology, shall be further investigated.
- The proposed methodology does not consider time-dependent aspects, namely, resistance gains over time as well as deterioration mechanisms that affect concrete properties (e.g., carbonation, chloride ingress, creep, among others) and, ultimately, influence the whole service lifetime of structures. The extension of the proposed methodology to include such time-dependent effects requires the utilisation of a more advanced safety concept that enables a more robust characterisation of the basic variables involved through other distribution types (e.g., Weibull, Gamma). Such analysis requires the utilisation of advanced reliability-based methods, such as simulation methods, which can handle complex time-dependent approaches (e.g.,^4,14^).
- Data-driven approaches are employed in statistical models, utilising either experimental data or field measurements (e.g.,^62,63^). Machine learning techniques—as part of data-driven reliability approaches—can be applied to exploit the available wave of information in real time (if possible) to implement predictive maintenance or other countermeasures—and ultimately, to provide a more robust reliability prediction^64^. While the utilisation of data-driven approaches have become a subject of gradual interest and investigation, advanced approaches have been emerging for existing structures. Yet, further investigations should be conducted regarding the interconnection between the proposed methodology and the recent advancements in data-driven reliability research.

## Data Availability

All data generated and analysed during the current study are available from the corresponding author upon reasonable request.

## Appendix 1: Iterative procedure to determine the statistical parameters of the lognormal distribution

To determine the coefficient of variation (COV) and the standard deviation $\sigma$ of the lognormal distribution from given mean value and a 5 $\%$ fractile (quantile), an iterative procedure was considered. Let’s assume that the mean value ${f}_{cm}$ is known, for example, ${f}_{cm}$ = 38 MPa for concrete class C25/30 under water curing conditions (Table 8). The 5 $\%$ fractile is taken as ${f}_{ck}$ = 30 MPa for this concrete class. The objective is to find parameters of the lognormal distribution that reproduce these two statistical properties. For a Lognormally-distributed variable ${f}$, the relationship between the mean ${f}_{cm}$, the standard deviation $\sigma$ and the parameters for the underlying normal distribution—$\lambda$ (mean of the logarithmic variable) and $\xi$ (standard deviation of the logarithmic variable)^16^—is given as:

19 $$\begin{aligned} \sigma \, = \text {COV} \cdot {f}_{cm} \end{aligned}$$

with the parameter $\lambda$ and $\xi$ being determined as:

20 $$\begin{aligned} \lambda \,= & \text {ln} - \frac{\xi ^2}{2} \end{aligned}$$

21 $$\begin{aligned} \xi \,= & \sqrt{\text {ln} \left( \left( \frac{\sigma }{{f}_{cm}} \right) ^2 +1 \right) } \end{aligned}$$

Note that the parameters $\lambda$ and $\xi$ fully define the lognormal distribution. To identify a consistent pair of COV and $\sigma$, an iterative loop is performed:

1. Start with an initial estimate of the COV.
2. Compute the corresponding standard deviation $\sigma \, = \text {COV} \cdot {f}_{cm}$.
3. Calculate $\lambda$ and $\xi$ using Eqs. 19, 20 and 21.
4. Determine the 5 $\%$ quantile ${f}_{ck.calc}$ of the resulting lognormal distribution, using the inverse transformation of the normal distribution: ${f}_{ck.calc}$ = $\text {exp}$ ( $\sigma$ + ${z}_{0.05}$ · $\zeta$) with ${z}_{0.05}$ = – 1.645 being the 5 $\%$ quantile of the standard normal distribution.
5. Compare the calculated ${f}_{ck.calc}$ with the target value ${f}_{ck}$ = 30 MPa.
6. Adjust the COV and repeat the process until the calculated quantile matches the target value within an acceptable tolerance.

Once convergence is achieved, the resulting values of COV and $\sigma$ are consistent with the specified mean strength ${f}_{cm}$ and 5 $\%$ characteristic strength ${f}_{ck}$.
